# Refined Node Energy Consumption Modeling in a LoRaWAN Network

**DOI:** 10.3390/s21196398

**Published:** 2021-09-25

**Authors:** Sébastien Maudet, Guillaume Andrieux, Romain Chevillon, Jean-François Diouris

**Affiliations:** Université de Nantes, CNRS, IETR UMR 6164, F-85000 La Roche-sur-Yon, France; guillaume.andrieux@univ-nantes.fr (G.A.); romain.chevillon@univ-nantes.fr (R.C.); jean-francois.diouris@univ-nantes.fr (J.-F.D.)

**Keywords:** energy consumption, Internet of Things (IoT), LoRa, LoRaWAN, LPWAN, wireless sensor network (WSN)

## Abstract

LPWAN technologies such as LoRa are widely used for the deployment of IoT applications, in particular for use cases requiring wide coverage and low energy consumption. To minimize the maintenance cost, which can become significant when the number of sensors deployed is large, it is essential to optimize the lifetime of nodes, which remains an important research topic. For this reason, it is necessary that it is based on a fine energy consumption model. Unfortunately, many existing consumption models do not take into account the specifications of the LoRaWAN protocol. In this paper, a refined energy consumption model based on in-situ measurements is provided for a LoRaWAN node. This improved model takes into account the number of nodes in the network, the collision probability that depends on the density of sensors, and the number of retransmissions. Results show the influence of the number of nodes in a LoRaWAN network on the energy consumption of a node and demonstrate that the number of sensors that can be integrated into a LoRaWAN network is limited due to the probability of collision.

## 1. Introduction

The integration of sensor networks into IoT solutions has opened the way for new services. In the very near future, many objects will be connected to improve the daily life of users through direct interactions with their environment [1,2]. Examples of applications are numerous, from smart city management [3] to process monitoring in Industry 4.0 [4], including the improvement of agricultural production [5,6], as well as the prevention of natural disasters [7] and the protection of ecosystems [8].

### 1.1. Related Works

#### 1.1.1. General LoRa Works

The LoRa technology still arouses curiosity and interest in the scientific community. This solution has been deeply studied and analyzed in order to validate use cases, to check the operating limits [9], to compare the technology with its peers or to propose improvement paths [10,11,12]. In [13], the authors measure the performance of a LoRa network in a real-world sensor-based monitoring environment. To verify the quality of service provided by the network, they generate data traffic to extract various metrics such as packet error rate (PER), received signal strength indicator (RSSI) and signal-to-noise ratio (SNR). Data rates and transmission times are determined in [14] to analyze transmission parameters. The authors of [15] define the maximum number of end devices (EDs) and their spatial distribution around a single gateway (GW). The maximum range of a LoRa modulated signal for fixed and mobile objects is measured in [16] under different transmission conditions. In [17], the authors highlight the 6720 possible combinations of transmission parameters. They propose an algorithm to maximize performance and energy consumption. In [18], a standalone LoRa sensor is developed to measure the transmission efficiency for different situations. All these studies validate the use of LoRa technology in IoT applications and the fact that transmission characteristics have a significant impact on the network’s behavior and reliability. In [19,20,21], the authors make a comparison between LoRa and other IoT technologies. They conclude that the advantages of the LoRa protocol are the lifetime, the cost and the capacity, and the disadvantages are the quality of service, the latency and the reliability. Finally, different surveys on the LoRa technology or LPWANs in general are introduced in [22,23,24]. These works review the state of the art and discuss future research challenges.

#### 1.1.2. Energy Consumption in LoRa

The energy criteria have been addressed by many works in the literature. In [25], the authors present an energy model in which the consumption of each element of a LoRa sensor has been analyzed (measurement stage, processing stage, radio interface). Transmission characteristics are chosen and compared with the aim of achieving energy autonomy. However, their model does not take into account transmission errors and collisions that depend on the density of sensors in the network. A Markov chain is used in [26] to model the consumption and the optimization of a LoRa node powered by a battery-free power source. They demonstrate the possibility of operating a sensor with an unreliable power source by optimizing operating properties (packet size, time between transmissions, optimization of the power circuit). A tool is suggested in [27] to compare IoT communication protocols. The solution calculates the energy consumption for a given protocol according to the operating time and the power required for a use case. Their modeling is intended to be generalist and does not fully consider the operating and modulation parameters of LoRa technology. An analytical model of LoRa node energy consumption is provided in [28]. The average current consumed by the sensor in a communication phase is estimated considering a given collision probability, a fixed transmission error rate and retransmissions. This work also introduces measurements carried out in a laboratory. However, their model does not take into account the environment, and in particular the density of devices in the network that will have a strong impact on the number of collisions. Other works use energy consumption as a metric to study scalability or reliability. In [29], the authors develop an experiment-based model to build a simulator. In [30], the authors develop a LoRa simulator in OMNET++ to evaluate the performance of a network using an Adaptive Data Rate (ADR) mechanism. They propose solutions that significantly increase the energy efficiency in dense networks. In [31], the authors introduce a cross layer simulation framework to evaluate and optimize the energy consumption of a device under realistic conditions. These works are based on simulations that do not really take into account all the recommendations of LoRaWAN specifications. Moreover, these studies consider the whole network’s energy consumption, whereas our analytical model focuses on the energy consumption of a LoRaWAN node in its environment.

### 1.2. Contributions and Organization

Our original works consider the energy consumption modeling of a LoRaWAN node in its environment. When the number of nodes increases in the network, the energy consumed by a node increases and its lifetime is reduced. The main contributions of our paper are as follows :

- In-situ measurements are made through a complete LoRaWAN testbed including the network server. The results allow us to characterize the consumed energy and the time spent for each LoRaWAN communication step;
- A refined energy consumption model for a LoRaWAN node is presented. This model takes into account the in-situ measurements, the number of nodes in the network, the collision probability that depends on the density of sensors, and the number of retransmissions.

This article is organized as follows. The LoRa protocol and LoRaWAN framework are described in Section 2. In-situ measurements that permit the evaluation of the LoRa node energy consumption are presented in Section 3. Section 4 introduces collision probability, transmission error rate and number of retransmissions. Then, the energy consumption model is presented, depending on the number of nodes in a LoRaWAN network. Finally, we draw a conclusion in Section 5.

## 2. LoRaWAN Overview

### 2.1. LoRa/LoRaWAN Architecture

**Figure 1:**
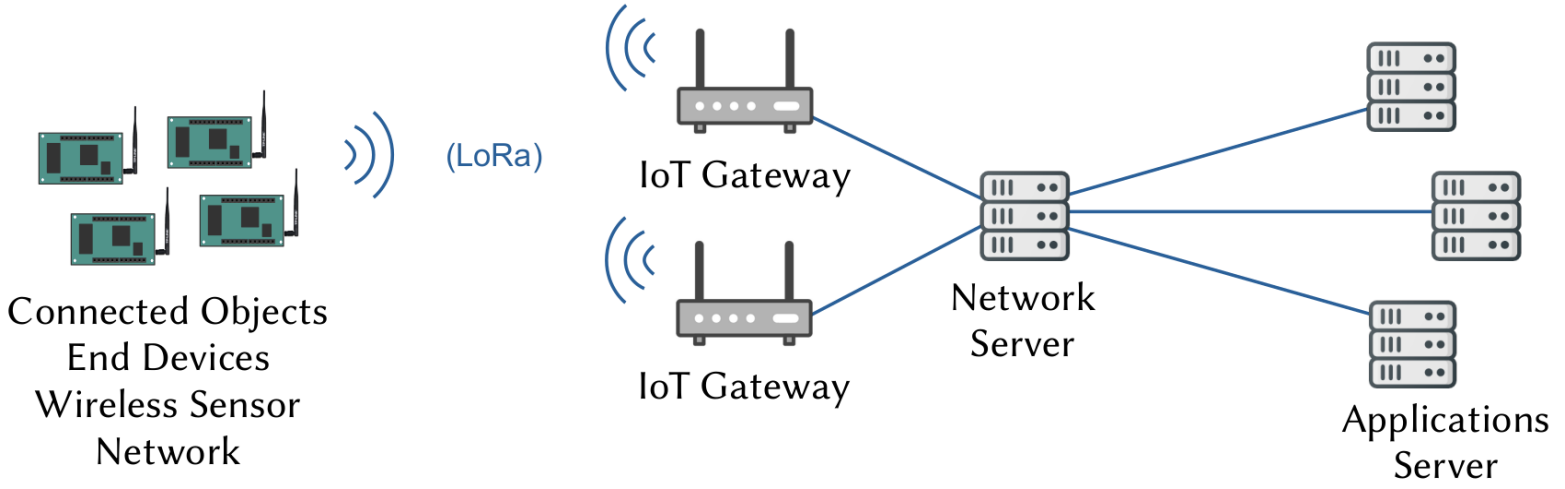
IoT network architecture.

The architecture and operations performed by an IoT network using LoRa/LoRaWAN technology have been described in detail [1,2,23,24,32,33]. The topology is star of stars, as shown in Figure 1. IoT nodes or End Devices (EDs) form the sensor network and are positioned as closely as possible to the physical environment. They interact with the medium and transmit data to one or more GWs using a wireless link with LoRa modulation. GWs perform a protocol transition in order to facilitate the communication from the wireless sensor network to the network server. Application servers store data and offer new services to the users. The network server administrates and secures the data transmission from the EDs.

**Figure 2:**
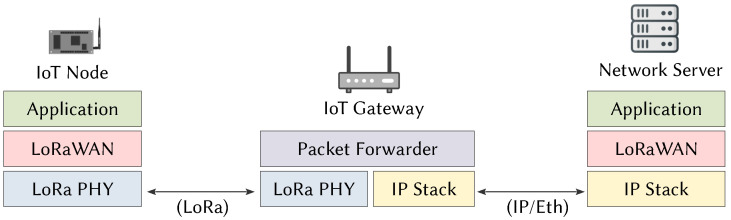
OSI modeling of the LoRaWAN protocol.

The LoRa/LoRaWAN solution fulfills the functions of the PHY and MAC layers of the OSI model. The LoRa physical layer is in charge of the modulation and the adaptation of the signal to the media. The LoRaWAN link layer provides an Aloha-type media access method. Figure 2 shows the deployment of LoRa technology in each network element. EDs encapsulate the data to be transmitted to application servers using both LoRa/LoRaWAN layers. The GW forwards the packet by re-encapsulating the LoRaWAN layer and data into an IP stack more suitable for communication with servers. The network server uses LoRaWAN and frame layers to acknowledge and manage the data transmissions of the wireless sensor network.

Nodes can communicate with one or more GWs. Packets from the various GWs are filtered by the network server before sending the data to application servers.

### 2.2. LoRa Modulation

The LoRa physical layer is open source and supports two-way communications using Chirp Spread Spectrum (CSS) modulation in unlicensed sub-GHz frequency bands. The spectrum is spread, that is, the modulated signal occupies a higher bandwidth than the source signal. This modulation technique has the advantage of making the signal more resistant to noise and interference [34,35,36], but also promotes orthogonal separation of signals transmitted on the same channel. The data rate is greatly reduced, which results in a significant increase in range. The main features of this protocol are summarized in Table 1.

**Figure 3:**
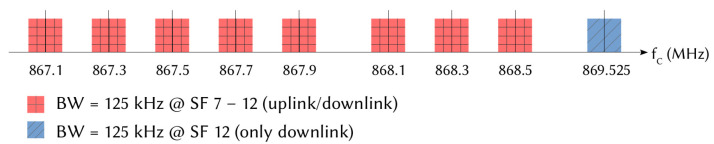
Example of LoRa channels used in Europe.

LoRa equipment operates in a sub-GHz frequency band, depending on the regional regulations [37] and the network settings. Each channel occupies a bandwidth (BW) of 125, 250 or 500 kHz. In Europe, two frequency bands are allowed: EU863–870 and EU433. Figure 3 shows an example of division for the 863–870 MHz band. In this case, the LoRa node must support at least 868.1 MHz, 868.3 MHz and 868.5 MHz channels, which are necessary for the joining process. Other channels can be used for uplink and downlink depending on the configuration of the GW, except for the 869.525 MHz channel, which is reserved for the downlink transmission of messages from the GW to the ED.

**Figure 4:**
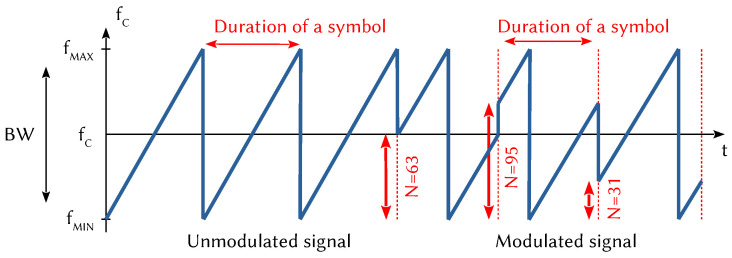
LoRa CSS modulation.

During the development of a frame, the transmitter carries out a number of processing operations to improve the robustness of the signal and to increase the sensitivity of the receiver. In particular, an Error-Correcting Code adds one to four cyclic redundancy bits to each 4-bit packet of data. The resulting binary data blocks are then grouped together to form SF-bit symbols (SF denotes the spreading factor) that modulate an up-chirp signal by frequency hopping [34,35,36]. Figure 4 shows an example of this modulation, where $f_{c}$ is the carrier frequency. On the left, it is an unmodulated sinusoidal up-chirp signal whose frequency varies proportionally over time. When it reaches the high frequency $f_{max}=f_{c}+\frac{\mathrm{BW}}{2}$, it switches to the low frequency $f_{min}=f_{c}-\frac{\mathrm{BW}}{2}$. On the right, several symbols have been modulated, each containing SF bits (the number of possible value is $2^{\mathrm{SF}}$). In this example, the spreading factor SF is 7 and the numerical value can range from 0 to 127. Downlink frames transmitted by the GWs to the EDs are done using inverse polarization [38].

### 2.3. Data Rate

The data rate is directly derived from the previous analysis and is given by:

(1) $$\begin{array}{c} R_{b}=\mathrm{SF}\frac{\mathrm{BW}}{2^{\mathrm{SF}}}\mathrm{CR}, \end{array}$$

where $R_{b}$, SF, BW and CR denote the data rate (in bits per second), the spreading factor, the bandwidth of the channel and the error code correction (from 4/5 to 4/8), respectively. The LoRa node adapts this coding rate according to the distance to the GW, but also when it resumes transmission after a failure. Table 2 presents the operating modes defined and implemented in LoRaWAN equipment [39]. Each mode, called Data Rate (DR5 to DR0), is a combination of modulation parameters chosen in order to reduce the data rate. The chosen spreading factor ${\mathrm{SF}}_{i}$ depends on the mode DRi. The LoRa/LoRaWAN specifications do not enforce a particular DR management strategy but recommend the following usage. A node close to the GW transmits in DR5 mode. If the transmission fails, it retransmits the same frame in DR5 mode and then decrements the DR every two retransmissions. A node further away transmits its first frame with a smaller DR and decrements the DR in the same way as every other retransmission [39].

### 2.4. Pathloss Model

Various pathloss models are taken into account in the literature. The authors of [30,31,40] use a log-distance pathloss model that includes small-scale fading. This model is well adapted for dense environments. The authors of [41] provide models for deployment in urban, forest and/or coastal environments. In [42], the authors propose a model for indoor environments and in [43,44], the authors use models for rural and urban environments based on field measurements. These models are more or less complex and can have a significant impact on the results. In order to simplify and facilitate the generalization of our energy model, we have chosen to use a simple pathloss model. According to European regulations, the maximum power allowed for transmission is set to 25 mW or 14 dBm [37]. In these conditions, for our considered model, the distance from the sensor to the GW is given by:

(2) $$d={\left[{\left(\frac{c}{4\pi f}\right)}^{2}\frac{P_{\mathrm{TA}}}{P_{\mathrm{RA}}}\right]}^{\frac{1}{n}},$$

where *d*, *c*, *f*, $P_{\mathrm{TA}}$, $P_{\mathrm{RA}}$ and *n* denote the distance between the considered ED and the GW, the velocity of the electromagnetic waves, the transmission frequency, the transmitted power, the received power and the path loss exponent, respectively. The values of the path loss exponent are n=2 in free space, n=3 in urban environments and n=6 in highly obstructed environments. In our study, the path loss exponent is considered equal to 3 and $P_{\mathrm{RA}}$ is determined with the SX1272 circuit datasheet [45]. In our energy model, we determine the data rate used by the sensor during its first transmission attempt using (2) by first computing $P_{\mathrm{RA}}$, which allows us to determine ${\mathrm{SF}}_{i}$ then DRi with Table 3.

### 2.5. LoRaWAN Media Access Method

**Figure 5:**
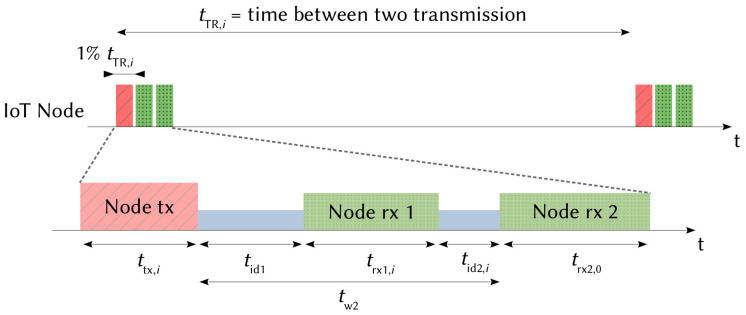
LoRaWAN class A transmission scheduling.

LoRaWAN is a solution based on an Aloha-type media access method that uses LoRa modulation on the physical layer. In order to share access to the media, European regulations impose the use of a duty cycle limitation or the so called “Listen Before Talk” transmission management. The LoRaWAN specification only implements the first solution with a duty cycle that must be less than 1%. Three classes of operation—A, B and C—are defined. In this article, we focus only on class A, which is the most energy-efficient and the only one mandatorily implemented in an equipment [37,39]. This operation mode is represented in Figure 5.

When a LoRa node has data to send, it randomly selects a channel and opens a transmission window (tx) followed by two reception windows (rx1 and rx2) used by the network server to acknowledge messages and administrate the communication. The sensor uses the same operating mode DRi and the same channel to receive the acknowledgment in the first reception window as those used during the transmission window. Then, a preconfigured and fixed operating mode and channel are used in the second window [37,39], which are DR0 and 869.525 MHz in Europe. The duration of the transmission ($t_{\mathrm{tx},i}$) and the duration of the first reception windows ($t_{\mathrm{rx}1,i}$) depend on the DR mode used; *i* represents the index of the DR and all the transmission parameters, as shown in Table 2. The duration of the second reception window ($t_{\mathrm{rx}2,0}$) depends on the DR0, where i=0. The LoRa node will only open the second reception window if it has not received an acknowledgment in the first window. In order to minimize power consumption, the LoRaWAN specification recommends leaving the equipment in the receive state only if a preamble is detected at the beginning of one of the receive windows. Otherwise, the equipment returns to the idle state after a preamble detection time. The duration of a reception window can therefore take two values given by:

(3) $$\begin{array}{c} t_{\mathrm{rx}1,i}=\left\{\begin{array}{cc} t_{\mathrm{ack},i} & \mathrm{if a preambule is detected in rx}1.\\ t_{\mathrm{pre},i} & \mathrm{if no preambule are detected in rx}1. \end{array}\right. \end{array}$$

(4) $$\begin{array}{c} t_{\mathrm{rx}2,0}=\left\{\begin{array}{cc} t_{\mathrm{ack},0} & \mathrm{if a preambule is detected in rx}2.\\ t_{\mathrm{pre},0} & \mathrm{if no preambule are detected in rx}2. \end{array}\right. \end{array}$$

Transmission and reception slots are spaced by two idle times named $t_{\mathrm{id}1}$ and $t_{\mathrm{id}2,i}$. $t_{\mathrm{id}1}$ can typically take a value between 1 and 15 s (classically, $t_{\mathrm{id}1}=1\;s$). $t_{w2}$ represents the time between the end of the transmission and the beginning of the second reception window ($t_{w2}=t_{\mathrm{id}1}+t_{\mathrm{rx}1,i}+t_{\mathrm{id}2,i}$). It can take a value between 2 and 16 s (classically, $t_{w2}=2\;s$) [37,39]. In our study, and respecting the recommendations of the specifications, we consider that all messages must be confirmed and that the network server only sends acknowledgments, which is the case in a normal operating LoRaWAN network.

### 2.6. Payload

**Figure 6:**
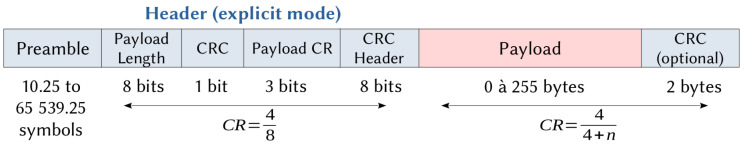
LoRa Physical Protocol Data Unit.

**Figure 7:**
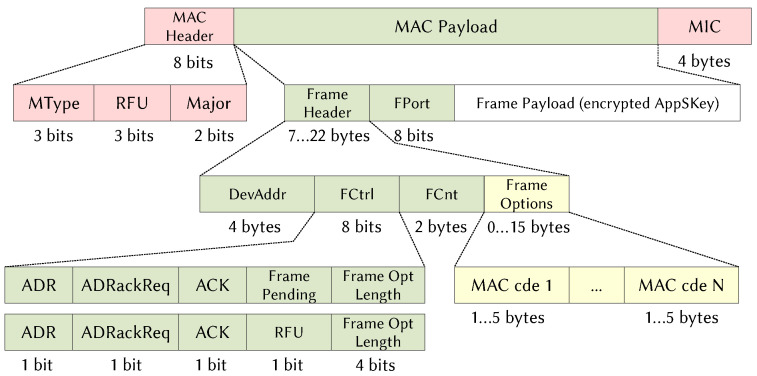
LoRaWAN MAC Protocol Data Unit.

Figure 6 shows a LoRa physical layer frame. It contains a header, a payload and an optional 16-bit CRC calculated on the payload. The physical header begins with the preamble, which is required for receiver synchronization. In Europe, the size (NP) is eight up-chirp symbols, followed by two symbols containing the network identifier, called the synchronization word (SW), and 2.25 down-chirp symbols. The rest of the header is not required in implicit mode. In explicit mode, an 8-bit field defines the length of the payload, a CRC bit indicates if the optional CRC is presented at the end of the frame, a 3-bit field contains the CR value used to encode the payload, and an 8-bit CRC is calculated on the header. The payload, from 0 to 255 bytes, contains the MAC header and the MAC payload as shown in Figure 7.

Three fields comprise the MAC header: the MAC type, the RFU for a future use, and the major field that indicates the protocol version. The 4-byte MIC field is used to check data integrity. In the MAC payload, the Frame header contains information about the data. FPort, DevAddr, FCtrl, FCnt, FOpts are, respectively, the service port, the address of the ED, information about the transmission, the frame counter and MAC commands transmitted by the network server to manage the communication of an ED. For the rest of the study, and for the sake of simplification, we consider a constant load of 50 bytes for the data frames and a physical explicit mode header. According to the standard, ACK frames are load-free at the application layer and there is no CRC at the physical level. The considered elements are summarized in Table 4.

Based on Figure 6 and Figure 7, the physical payload is given by:

(5) $$\begin{array}{cc} \; & {\mathrm{PL}}_{\mathrm{data}}=H2+H3+\mathrm{FPayload},\\ \; & {\mathrm{PL}}_{\mathrm{ack}}=H2+H3, \end{array}$$

where ${\mathrm{PL}}_{\mathrm{data}}$, ${\mathrm{PL}}_{\mathrm{ack}}$, H2, H3 and FPayload denote, respectively, the size of the physical layer payload for a data frame, the size of the physical layer payload for an ACK frame, the size of the MAC header and the MIC, the size of the application header and the size of the data frame payload. From these equations, the number of symbols contained in the data and the ACK frames are determined in (6), where H1, CR, CRC and DE denote, respectively, the size of the physical header, the coding rate, the size of the CRC used for the data frame and the low data rate optimization factor. Note that DE=1 when ${\mathrm{SF}}_{i}=11$ or ${\mathrm{SF}}_{i}=12$, 0 otherwise [39].

(6) $$\begin{array}{cc} \; & N_{\mathrm{data},i}=8+\max\left(\mathrm{ceil}\left(\frac{H1+8\cdot {\mathrm{PL}}_{\mathrm{data}}+\mathrm{CRC}+8-4\cdot {\mathrm{SF}}_{i}}{4\cdot ({\mathrm{SF}}_{i}-2\cdot \mathrm{DE})}\right)\frac{4}{{\mathrm{CR}}_{i}},0\right),\\ \; & N_{\mathrm{ack},i}=8+\max\left(\mathrm{ceil}\left(\frac{H1+8\cdot {\mathrm{PL}}_{\mathrm{ack}}+8-4\cdot {\mathrm{SF}}_{i}}{4\cdot ({\mathrm{SF}}_{i}-2\cdot \mathrm{DE})}\right)\frac{4}{{\mathrm{CR}}_{i}},0\right). \end{array}$$

### 2.7. Time on Air

As seen in Figure 5, a LoRa device has different states during a communication phase. Times corresponding to data processing, waking up and switching off the radio interface are incompressible, do not change and depend solely on the electronic circuits implemented on the board. Transmission and reception times ($t_{\mathrm{tx},i}$, $t_{\mathrm{rx}1,i}$ and $t_{\mathrm{rx}2,0}$) vary according to characteristics of the LoRa modulation that correspond to the time on air used to transmit a data frame and to receive an ACK frame. $t_{\mathrm{tx},i}$, $t_{\mathrm{ack},i}$ and $t_{\mathrm{pre},i}$ are obtained by the following equations:

(7) $$\begin{array}{cc} \; & t_{\mathrm{tx},i}=\left(\mathrm{NP}+\mathrm{SW}+2.25+N_{\mathrm{data},i}\right)\cdot T_{S,i},\\ \; & t_{\mathrm{ack},i}=\left(\mathrm{NP}+\mathrm{SW}+2.25+N_{\mathrm{ack},i}\right)\cdot T_{S,i},\\ \; & t_{\mathrm{pre},i}=\mathrm{NP}\cdot T_{S,i}, \end{array}$$

where $T_{S,i}=\frac{2^{{\mathrm{SF}}_{i}}}{\mathrm{BW}}$ is the duration of a symbol.

## 3. LoRa Node Energy Consumption

### 3.1. Theoretical Analysis of Energy Consumption

With the elements obtained in the previous section, the first model of the energy consumption is proposed without taking into account transmission errors and collisions. From Figure 5, the energy consumed by a node that receives the ACK in the first window is given by:

(8) $$\begin{array}{c} E_{\mathrm{bit}}=\frac{P_{\mathrm{tx}}\cdot t_{\mathrm{tx},i}+P_{\mathrm{id}}\cdot t_{\mathrm{id}1}+P_{\mathrm{rx}}\cdot t_{\mathrm{ack},i}}{8\cdot \mathrm{FPayload}}, \end{array}$$

where $E_{\mathrm{bit}}$, $P_{\mathrm{tx}}$, $P_{\mathrm{rx}}$, $P_{\mathrm{id}}$ are the average energy consumed by the node for each useful bit transmitted, the power consumed by the node during the transmission, the power consumed by the node during the reception and the power consumed by the node in the idle state, respectively. FPayload is multiplied by eight to obtain it in bits.

Similarly, the energy consumed by a node that receives the ACK in the second window is given by:

(9) $$\begin{array}{c} E_{\mathrm{bit}}=\frac{P_{\mathrm{tx}}\cdot t_{\mathrm{tx},i}+P_{\mathrm{id}}\cdot t_{\mathrm{id}1}+P_{\mathrm{rx}}\cdot t_{\mathrm{pre},i}+P_{\mathrm{id}}\cdot t_{\mathrm{id}2,i}+P_{\mathrm{rx}}\cdot t_{\mathrm{ack},0}}{8\cdot \mathrm{FPayload}}. \end{array}$$

### 3.2. Measurement of Consumed Powers

#### 3.2.1. Current Measurement Protocol

**Figure 8:**
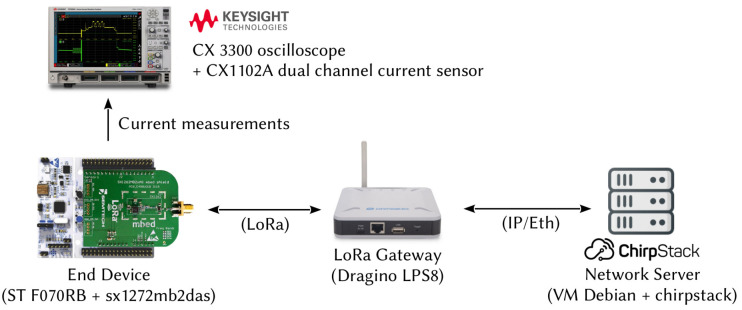
Scheme of the experimental testbed.

**Figure 9:**
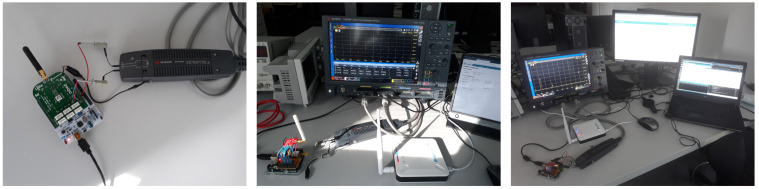
Experimental testbed to measure currents consumed by a LoRa shield of an IoT node.

The current consumed and the time spent by a LoRa circuit in the different operating states were measured. An end-to-end LoRaWAN communication between one sensor node and a network server was implemented. Figure 8 and Figure 9 show the testbed. The current analyzer was a Keysight CX3300 with a CX1102A dual channel current probe.

The sensor node was made of a Nucleo F070RB board equipped with an sx1272mb2das LoRa shield. The gateway was a Dragino LPS-8 and the network server was built around a Chirptack solution installed on a Debian OS. All the communications respected the LoRaWAN specifications. Authentication of the node to the network server was done with the ABP method for its simplicity. The end device transmitted random data of 50 bytes at regular intervals, respecting the duty cycle, by decreasing the DR at each transmission attempt. Several parameters used by the node were configured in the network server and transmitted to the end devices with MAC commands. The minimum and the maximum values of the DR were set to 0 and 5, respectively; the Rx2 slot was configured with DR = 0 on the channel at 869.525 MHz and the maximum power allowed was 14 dBm. For a new transmission attempt, the channel was selected randomly by the node and no offset was set for the ACK in the first reception window. Idle times $t_{\mathrm{id}1}$ and $t_{\mathrm{id}2,i}$ were configurable using the *RXTimingSetupReq* and *RXTimingSetupAns* MAC commands. We configured $t_{\mathrm{id}1}$ and $t_{w2}$ at the minimum value, $t_{\mathrm{id}1}$ = 1 s and $t_{w2}$ = 2 s. In order to avoid collisions, measurements were made with only one end device connected to the gateway.

#### 3.2.2. Current Measurement Results

**Figure 10:**
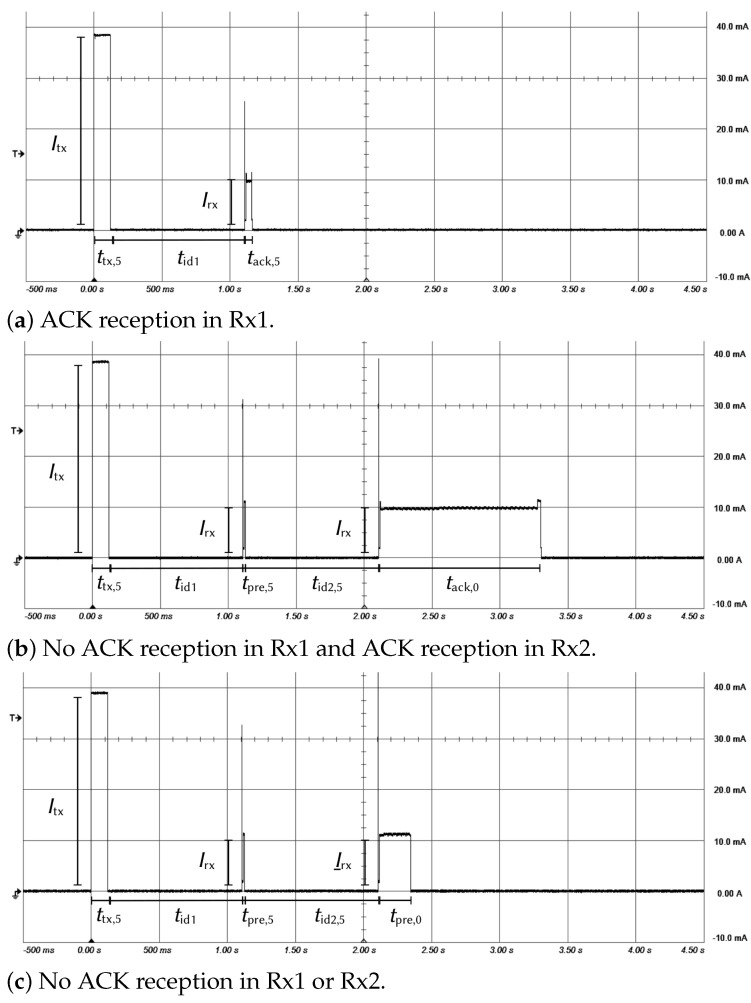
Measured current in various transmission phases ($P_{\mathrm{tx}}=14\;\mathrm{dBm}$, DR = 5).

Figure 10 shows the measurement results of the current consumed by the node in a communication phase when the ACK was received in the first reception window (a), when the ACK was not received in the first reception window but in the second reception window (b) and when no ACK was received by the node (c).

**Figure 11:**
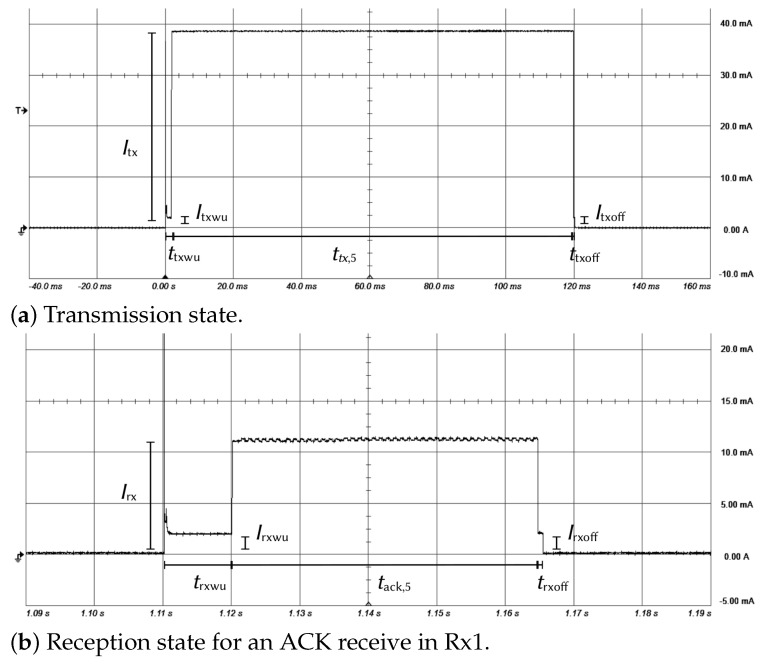
Measured current in transmission and reception states (DR = 5).

These figures show the measurement results for various operating states. In each case, the circuit begins by positioning itself in a transmission state followed by one or two idle and reception states. Transmission and reception durations ($t_{\mathrm{tx},i}$, $t_{\mathrm{rx}1,i}$, $t_{\mathrm{rx}2,0}$) depend on the DR mode used by the node and its location. If an ACK is received in the first receive window, the equipment does not open the second receive window (Figure 10a). If the network server does not send an acknowledgment or if it is not received by the node, the latter stays in the reception state for a time less than or equal to the duration of the preamble and open a second receive window (Figure 10b). Finally, if an ACK is not received in the second receive window, the node stays in a reception state only during a preamble (Figure 10c) and goes to sleep mode. Figure 11a details the moment when the sensor is in a transmission state and Figure 11b when the sensor is in a reception state. Each event starts with a wake-up and finishes with the standby of the circuit.

We performed acquisitions for all the DR modes and the results are summarized in Table 5 and Table 6. Table 5 contains currents drawn by the circuit in the different operating states during a communication phase. The theoretical values are those specified in the datasheet [45] and the experimental values are those obtained by the measurements. Current consumed for the wake-up ($I_{\mathrm{txwu}}$, $I_{\mathrm{rxwu}}$), for the standby ($I_{\mathrm{rxoff}}$, $I_{\mathrm{rxoff}}$) and for the reception ($I_{\mathrm{rx}}$) are constant, whatever the used DR. Current consumed by the circuit during the uplink phase ($I_{\mathrm{tx}}$) varies according to the transmission power. Measurement values are of the same order of magnitude as those specified in the datasheet [45], except for the idle current, which is 100 times higher. Table 5 and Table 6 also contain the time spent by the circuit in the different operating states. Time spent for the wake-up ($t_{\mathrm{txwu}}$, $t_{\mathrm{rxwu}}$) and for the standby ($t_{\mathrm{txoff}}$, $t_{\mathrm{rxoff}}$) are constant, whatever the used DR. Time spent to transmit data ($t_{\mathrm{tx},i}$), to receive an ACK ($t_{\mathrm{ack},i}$) and to listen to a preamble ($t_{\mathrm{pre},i}$) depend on the DR mode. Theoretical values obtained with (7) correspond to the values obtained in the laboratory and are presented in Table 6. In the rest of the article, the theoretical values are mainly used. The values obtained by measurements are only used when the theoretical values are not available.

## 4. Node Energy Consumption in a LoRaWAN Network

Energy studies must take into account the node environment. Errors and collisions depend on the network density and affect the energy consumption of the LoRaWAN network. In this section, we model these characteristics in order to propose a more refined node energy consumption model in such a network.

### 4.1. Error and Collision Probabilities

Communications using an Aloha access method can suffer from numerous collisions and transmission errors, especially with a large number of devices sharing the media. It is assumed that the messages are sent over the network using a Poisson process [1]. Then, the collision probability of data transmissions is given by:

(10) $$\begin{array}{cc} \; & p_{c_{\mathrm{data},i}}=1-e^{-2\cdot N_{\mathrm{sta}}\cdot p_{i}\cdot \lambda \cdot t_{\mathrm{tx},i}}, \end{array}$$

where $p_{c_{\mathrm{data},i}}$, $N_{\mathrm{sta}}$, $p_{i}$ and λ denote the probability of having a collision in the transmission of a data frame, the number of EDs in the network using the same channel, the probability that a sensor uses the SF with index *i* and the packet generation rate for an ED, respectively. Note that the presence of “2” in the exponent reflects the fact that a frame is vulnerable over a time that is twice as long as its duration. The product $N_{\mathrm{sta}}\cdot p_{i}\cdot \lambda$ represents the Poisson process intensity. In our study, we consider that λ is related to the duty cycle imposed by the European standard, that is, a value of 1% related to the channel occupancy time. The duty cycle dc and λ are given by:

(11) $$\begin{array}{cc} \; & \mathrm{dc}=\frac{t_{\mathrm{tx},i}}{t_{\mathrm{TR}}},\\ \; & \lambda =\frac{1}{N_{P}\cdot t_{\mathrm{TR}}}, \end{array}$$

where $t_{\mathrm{TR}}$ and $N_{P}$ denote the time between two transmissions and the number of packets in the ED, respectively. If the buffer of the ED is always full, we consider that $N_{P}=1$. The density of sensors for each SF is summarized in Table 7, from the works explained in [1,38,46].

Regarding collisions in the transmission of an ACK frame, the synchronization of transmissions at the network server (and the GW) means that there is no collision between the different ACK frames. We have seen previously that the sensor uses the same operating mode DRi and the same channel to receive the acknowledgment in the first reception window as those used during the transmission window. However, the LoRaWAN specifications require devices to reverse the IQ polarity for downlink messages compared to uplink messages [37,39]. Moreover, this assertion was verified during the measurements. Therefore, we can consider that there is no collision between ACK frames and data frames.

The transmission error probability for a CSS type modulation is given by ([28,47]):

(12) $$\begin{array}{cc} \; & p_{e_{\mathrm{data}},i}=1-{\left(1-Q\left(\frac{\log_{12}\left({\mathrm{SF}}_{i}\right)}{\sqrt{2}}\frac{E_{b}}{N_{0}}\right)\right)}^{\frac{1}{\mathrm{CR}}\cdot \left(N_{\mathrm{data},i}-H1\right)},\\ \; & p_{e_{\mathrm{ack}},i}=1-{\left(1-Q\left(\frac{\log_{12}\left({\mathrm{SF}}_{i}\right)}{\sqrt{2}}\frac{E_{b}}{N_{0}}\right)\right)}^{\frac{1}{\mathrm{CR}}\cdot \left(N_{\mathrm{ack},i}-H1\right)}, \end{array}$$

where $p_{e_{\mathrm{data},i}}$, $p_{e_{\mathrm{ack},i}}$, $\frac{E_{b}}{N_{0}}$, $N_{\mathrm{data},i}$, $N_{\mathrm{ack},i}$ denote the probability of having an error in the reception of a data frame, the probability of having an error in the reception of an ACK frame, the signal-to-noise ratio and the frame lengths determined by (6).

Subsequently, the probability of successful transmission for a data point and an ACK frame can be given by:

(13) $$\begin{array}{cc} p_{\mathrm{data},i}= & \left(1-p_{e_{\mathrm{data},i}}\right)\cdot \left(1-p_{c_{\mathrm{data},i}}\right)\\ = & {\left(1-Q\left(\frac{\log_{12}\left({\mathrm{SF}}_{i}\right)}{\sqrt{2}}\frac{E_{b}}{N_{0}}\right)\right)}^{\frac{1}{\mathrm{CR}}\cdot \left(N_{\mathrm{data},i}-H1\right)}\times e^{-2\cdot N_{\mathrm{sta}}\cdot p_{i}\cdot \lambda \cdot t_{\mathrm{tx},i}},\\ p_{\mathrm{ack},i}= & 1-p_{e_{\mathrm{ack},i}}\\ = & {\left(1-Q\left(\frac{\log_{12}\left({\mathrm{SF}}_{i}\right)}{\sqrt{2}}\frac{E_{b}}{N_{0}}\right)\right)}^{\frac{1}{\mathrm{CR}}\cdot \left(N_{\mathrm{ack},i}-H1\right)}. \end{array}$$

### 4.2. Node Energy Consumption for One Transmission

#### 4.2.1. Theoretical Analysis

**Figure 12:**
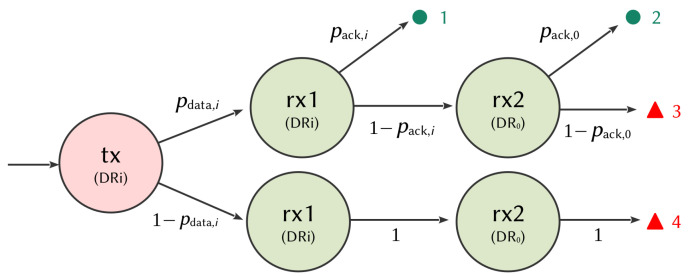
State chart of a LoRa node for one transmission.

**Figure 13:**
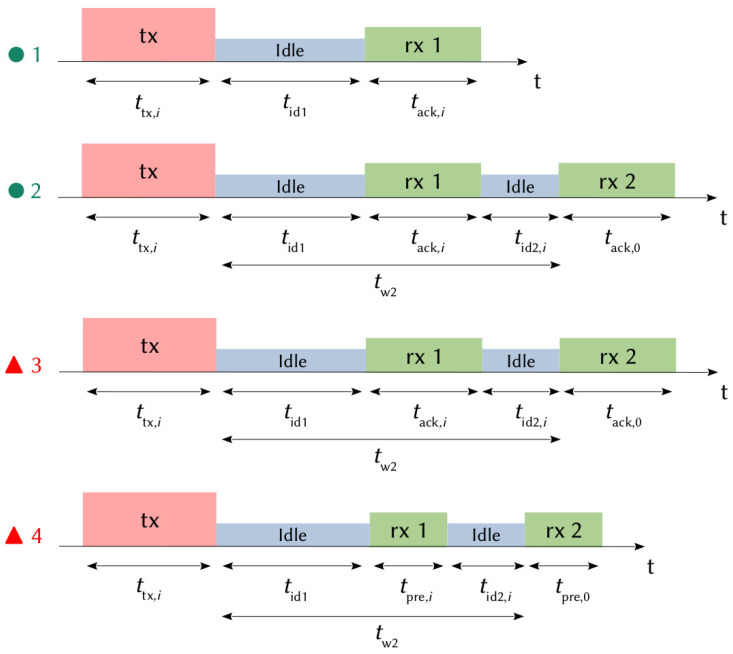
LoRa node state evolution for one transmission.

Figure 12 shows the state graph of a transmission. First, the ED transmits its data. Four different situations are possible after the transmission of the data, as depicted in Figure 13:

- ● 1: the gateway successfully receives the data, then the sensor receives an acknowledgment in the first window, with a probability $p_{\mathrm{data},i}\cdot p_{\mathrm{ack},i}$;
- ● 2: the gateway successfully receives the data, then the sensor does not receive an acknowledgment in the first window (with a probability $p_{\mathrm{data},i}\cdot \left(1-p_{\mathrm{ack},i}\right)$), opens the second reception window following the failure in the first, then correctly receives the acknowledgment;
- ▲ 3: the gateway successfully receives the data, then the sensor does not receive an acknowledgment in the first window (with a probability $p_{\mathrm{data},i}\cdot \left(1-p_{\mathrm{ack},i}\right)$), opens the second reception window following the failure in the first, then an error occurs again, causing a reception failure in the second window;
- ▲ 4: the gateway does not receive the data (with a probability $1-p_{\mathrm{data},i}$). The node is not aware of it, so it opens the two reception slots (rx1 and rx2) for a duration, allowing it to only receive the preamble of an ACK frame.

In the case of a successful transmission (● 1 and ● 2), the node goes into the sleep state for a time, respecting the duty cycle. In the case of a transmission failure, (▲ 3 and ▲ 4), the node goes into a sleep state and gets ready for a new attempt on another channel. In the case of transmission error, we consider that the node listens to the entire frame but is unable to decode it. In the case of collision, we consider that the node only listens to the preamble detection time, as indicated in the specification [37,39]. A node is indeed able to read a collision frame if the SIR is higher than a few dB.

From Figure 12 and Figure 13, the energy consumed by a sensor for the four different situations is given by :

(14) $$\begin{array}{cc} E_{1,i}= & E_{\mathrm{tx},i}+E_{\mathrm{id}1}+E_{\mathrm{rx}1,i}=P_{\mathrm{tx}}\cdot t_{\mathrm{tx},i}+P_{\mathrm{id}}\cdot t_{\mathrm{id}1}+P_{\mathrm{rx}}\cdot t_{\mathrm{ack},i} \end{array}$$

(15) $$\begin{array}{cc} E_{2,i}= & E_{\mathrm{tx},i}+E_{\mathrm{id}1}+E_{\mathrm{rx}1,i}+E_{\mathrm{id}2,i}+E_{\mathrm{rx}2,0}\\ = & P_{\mathrm{tx}}\cdot t_{\mathrm{tx},i}+P_{\mathrm{id}}\cdot \left(t_{\mathrm{id}1}+t_{\mathrm{id}2,i}\right)+P_{\mathrm{rx}}\cdot \left(t_{\mathrm{ack},i}+t_{\mathrm{ack},0}\right) \end{array}$$

(16) $$\begin{array}{cc} E_{3,i}= & E_{\mathrm{tx},i}+E_{\mathrm{id}1}+E_{\mathrm{rx}1,i}+E_{\mathrm{id}2,i}+E_{\mathrm{rx}2,0}\\ \; & P_{\mathrm{tx}}\cdot t_{\mathrm{tx},i}+P_{\mathrm{id}}\cdot \left(t_{\mathrm{id}1}+t_{\mathrm{id}2,i}\right)+P_{\mathrm{rx}}\cdot \left(t_{\mathrm{ack},i}+t_{\mathrm{ack},0}\right) \end{array}$$

(17) $$\begin{array}{cc} E_{4,i}= & E_{\mathrm{tx},i}+E_{\mathrm{id}1}+E_{\mathrm{rx}1,i}+E_{\mathrm{id}2,i}+E_{\mathrm{rx}2,0}\\ \; & P_{\mathrm{tx}}\cdot t_{\mathrm{tx},i}+P_{\mathrm{id}}\cdot \left(t_{\mathrm{id}1}+t_{\mathrm{id}2,i}\right)+P_{\mathrm{rx}}\cdot \left(t_{\mathrm{pre},i}+t_{\mathrm{pre},0}\right), \end{array}$$

where $E_{1,i}$, $E_{2,i}$, $E_{3,i}$, $E_{4,i}$ are the energy consumed by the sensor in the situations 1, 2, 3 and 4 of the state graph, respectively.

#### 4.2.2. Results and Discussions

**Figure 14:**
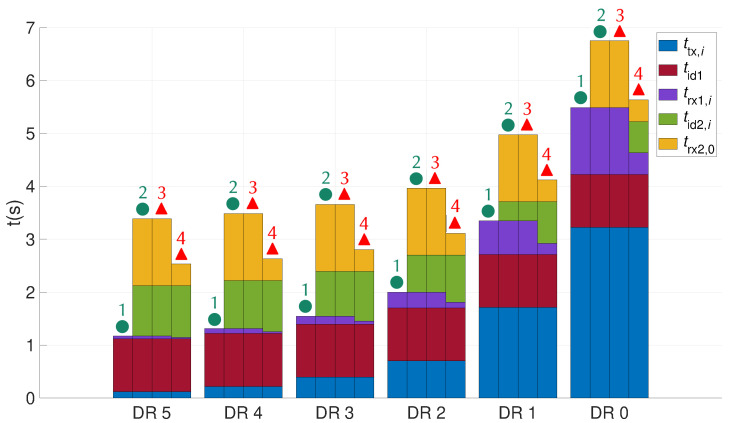
Spent time by a LoRa sensor in various operating states.

Figure 14 shows the time spent by a LoRa sensor in the different operating states for each path of the state graph (Figure 12 and Figure 13). For each DR, the first bar represents the sum of the times spent by the node when an ACK is successfully received in the first window ● 1. The second bar represents the sum of the times spent by the node when an ACK is successfully received in the second window ● 2. The third bar represents the sum of the times spent by the node when no ACK is received ▲ 3 and the fourth bar when the data is unsuccessfully received by the gateway ▲ 4.

Transmission time $t_{\mathrm{tx},i}$ and reception times $t_{\mathrm{rx}1,i}$, $t_{\mathrm{rx}2,0}$ vary according to the LoRa modulation characteristics established in (7) and the data frame size obtained with (5) and (6). Idle time $t_{\mathrm{id}1}$ is constant regardless of the used DR and $t_{\mathrm{id}2,i}$ varies according to the time spent by the node in the first reception window. Transmission time $t_{\mathrm{tx},i}$ and reception time $t_{\mathrm{rx}1,i}$ increase in a ratio of two when the DR mode decreases by one. The transmission time of a frame in DR0 is more than ten times larger than in DR4 and DR5 with values of 3.219 s, 218 ms and 118 ms, respectively. Proportionally, a sensor spends more time in an idle state when the DR is high and more time in the transmission and reception states when the DR is small. Table 8 contains the cumulative time spent by a node in all the situations. The wake-up time is almost tripled when the ED opens a second reception window for the DR5 and is multiplied by 1.25 for the DR0. It can be concluded that, in order to maximize sensor sleep time, communications with high DRs should be favored and the second reception window should be minimized.

**Figure 15:**
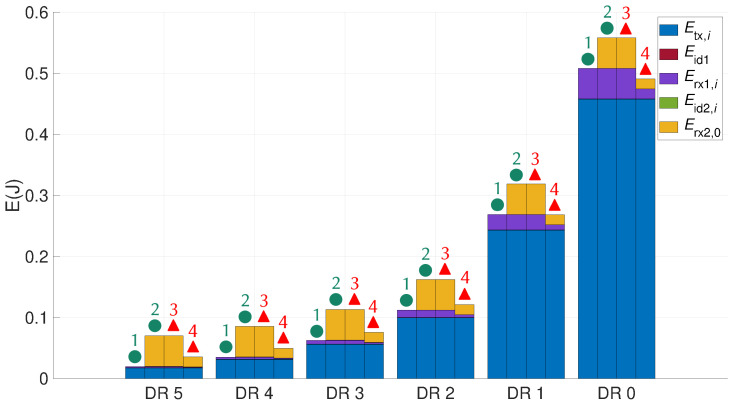
Total energy consumed by the node.

Figure 15 shows the cumulative energy consumed by a node in the different DRs, for each situation of the state graph (Figure 12 and Figure 13). This figure is obtained from (14), (15), (16), and (17). For each DR, the first bar represents the sum of the energy consumed by a node when an ACK is successfully received in the first window ● 1. The second bar represents the sum of the energy consumed by a node when an ACK is successfully received in the second window ● 2. The third bar represents the sum of the energy consumed by a node when no ACK is received ▲ 3 and the fourth bar when the data is unsuccessfully received by the gateway ▲ 4.

During a communication phase, energy is mainly consumed in the transmission state and then in the reception state. In an idle state, the energy is very low and $E_{\mathrm{id}1}$ is constant whatever the DR. Table 9 shows the sum of the energy consumed by a device in the different operating states for each path of the state graph. It can be noticed that the energy is multiplied by three when the ED opens the second reception window in DR5 and is increased by 10% when the equipment opens the second reception window in DR0. The impact of opening the second window on energy consumption is therefore greater for high DRs. Listening to the preamble in the receive windows saves between 30 and 80 mJ by putting the equipment into the sleep state earlier.

### 4.3. Node Energy Consumption with Multiple Retransmissions

#### 4.3.1. Theoretical Analysis

The LoRaWAN specifications recommend up to seven retransmissions in the case of failure, after which the packet is discarded. These attempts must be made on another randomly selected channel after a waiting time $t_{\mathrm{rep}}$. This waiting time corresponds to the RETRANSMIT TIMEOUT and is also randomly selected between 1 s and 3 s. It is possible to decrease the DR every two retransmissions from 5 to 0 and to remain at 0 for additional retransmissions. In our model, each retransmission is spaced by an average time $t_{\mathrm{rep}}$ = 2 s, considering that the probability of errors and collisions remains the same at each attempt (which is due to the selection of a new channel). Thus, the average node energy consumption for each situation (success and failure) is given by:

(18) $${\bar{E}}_{s,i}=E\left[E_{s,i}\right]=\frac{E_{1,i}\cdot p_{\mathrm{data},i}\cdot p_{\mathrm{ack},i}+E_{2,i}\cdot p_{\mathrm{data},i}\cdot p_{e_{\mathrm{ack}},i}\cdot p_{\mathrm{ack},0}}{p_{s,i}},$$

(19) $${\bar{E}}_{f,i}=E\left[E_{f,i}\right]=\frac{E_{3,i}\cdot p_{\mathrm{data},i}\cdot p_{e_{\mathrm{ack}},i}\cdot p_{e_{\mathrm{ack}},0}+E_{4,i}\cdot \left(1-p_{\mathrm{data},i}\right)}{p_{f,i}},$$

where ${\bar{E}}_{s,i}$, ${\bar{E}}_{f,i}$, $p_{s,i}$ and $p_{f,i}$ denote the average energy consumed by the node for a successful transmission (● 1, ● 2), the average energy consumed by the node for a failed transmission (▲ 3, ▲ 4), the probability of a successful transmission and the probability of a failed transmission, respectively. According to Figure 12, the probabilities $p_{s,i}$ and $p_{f,i}$ are determined by:

(20) $$p_{s,i}=p_{\mathrm{data},i}\cdot p_{\mathrm{ack},i}+p_{\mathrm{data},i}\cdot p_{e_{\mathrm{ack}},i}\cdot p_{\mathrm{ack},0},$$

(21) $$p_{f,i}=p_{\mathrm{data},i}\cdot p_{e_{\mathrm{ack}},i}\cdot p_{e_{\mathrm{ack}},0}+\left(1-p_{\mathrm{data},i}\right).$$

Then, the total energy consumed by a sensor depends on its distance from the gateway, the number of sensors in the network and the number of retransmissions allowed for the equipment. This calculation will be different if the number of retransmissions is even or odd and must take into account the double repetition of the transmission phase with the same DR, given by:

(22) $$\begin{array}{cc} \; & \alpha =\left\lceil \frac{\mathrm{NR}}{2}-1\right\rceil ,\\ \; & \beta =1+\mathrm{NR}-2\cdot \left\lceil \frac{\mathrm{NR}}{2}\right\rceil , \end{array}$$

where α∈N denotes the number of double retransmissions. β=1 if the number of transmissions (NR) is even and β=0 if NR is odd. The total energy consumed by a sensor is given as the sum of energies statistically consumed during a successful transmission and the sum of energies statistically consumed during a failed transmission. Then, the average total energy consumed by a node is given by:

(23) $$\begin{array}{cc} {\bar{E}}_{\mathrm{tot}}= & \sum_{n=0}^{\alpha }[\left({\bar{E}}_{s,\max({\mathrm{DR}}_{M}-n,0)}+\left\lceil \frac{n}{\max(\alpha ,1)}\right\rceil .\sum_{m=0}^{n-1}2\cdot {\bar{E}}_{f,\max({\mathrm{DR}}_{M}-m,0)}\right)\\ \; & \;\;\;\times (p_{s,\max({\mathrm{DR}}_{M}-n,0)}\cdot \prod_{m=0}^{n-1}p_{f,\max({\mathrm{DR}}_{M}-m,0)}^{2})\\ \; & \;+\mu \cdot ({\bar{E}}_{s,\max({\mathrm{DR}}_{M}-n,0)}+{\bar{E}}_{f,\max({\mathrm{DR}}_{M}-n,0)}+\left\lceil \frac{n}{\max(\alpha ,1)}\right\rceil .\sum_{m=0}^{n-1}2\cdot {\bar{E}}_{f,\max({\mathrm{DR}}_{M}-m,0)})\\ \; & \;\;\;\times (p_{s,\max({\mathrm{DR}}_{M}-n,0)}\cdot p_{f,\max({\mathrm{DR}}_{M}-n,0)}\cdot \prod_{m=0}^{n-1}p_{f,\max({\mathrm{DR}}_{M}-m,0)}^{2})]\\ \; & +(\beta \cdot {\bar{E}}_{f,\max({\mathrm{DR}}_{M}-\alpha ,0)}+{\bar{E}}_{f,\max({\mathrm{DR}}_{M}-\alpha ,0)}+\sum_{n=0}^{\alpha -1}2\cdot {\bar{E}}_{f,\max({\mathrm{DR}}_{M}-n,0)}).\\ \; & \;\;\times (p_{f,\max({\mathrm{DR}}_{M}-\alpha ,0)}^{\beta +1}\cdot \prod_{n=0}^{\alpha -1}p_{f,\max({\mathrm{DR}}_{M}-n,0)}^{2}), \end{array}$$

where ${\mathrm{DR}}_{M}$ and $\mu =\beta \vee \left\lceil \frac{\alpha -n}{\max(\alpha ,1)}\right\rceil$ denote the initial DR used by the sensor for its first transmission attempt (note that ${\mathrm{DR}}_{M}$ depends on the distance) and a coefficient that allows us to adjust the calculation to the number of phases in order to respect the operation established in the state graph (Figure 12), respectively.

#### 4.3.2. Results and Discussions

**Figure 16:**
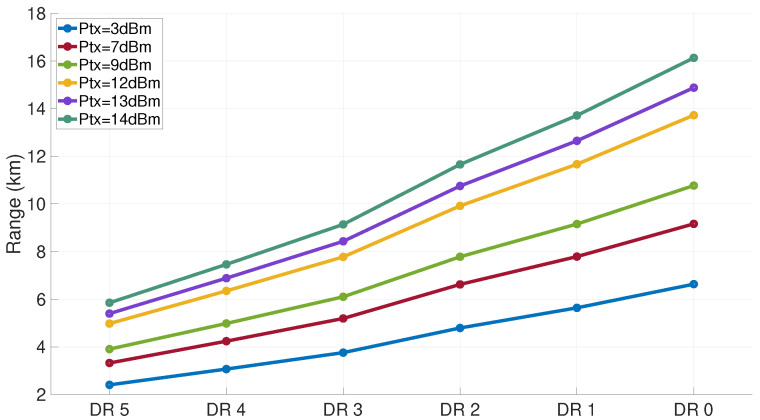
Maximum LoRa node range vs. DR and transmit power.

In this energy consumption model, (2) allows us to determine the SF used by the sensor for its first transmission attempt. Figure 16 shows the variation of this range as a function of the DR and the transmission power, for a loss exponent equal to three. The range of the signal increases when the DR mode decreases. A sensor located less than 4 km from the gateway can transmit using all the SF (note that the best efficiency is reached for DR5). A device located more than 10 km away can only use the SF12, corresponding to DR0. In our model, we compute the distance between the node and the gateway to determine the DR used by the node on its first transmission attempt, which corresponds to ${\mathrm{DR}}_{M}$ in (23).

The analytical model given in (23) corresponds to the average total node energy consumption. Then, the average energy consumption by useful bit can be defined by ${\bar{E}}_{\mathrm{bit}}=\frac{{\bar{E}}_{\mathrm{tot}}}{8\cdot \mathrm{FPayload}}$. Table 7 shows the probability that the considered node uses a specific SF. This probability is used to obtain the number of sensors using each DR. Then, the error and collision probabilities are obtained from the EDs’ distribution in the network. Thus, if the number of nodes in the network increases, the probabilities in Table 7 involve an increase of the number of nodes using the same DR, and then an increase of the collision probability. Subsequently, (10), (12) and (13) are used to determine the success and failure probabilities based on this distribution and, finally, (23) is used by varying different input parameters:

- the transmission power of the node;
- the load carried in the data frames;
- the number of allowed retransmissions;
- the distance between the sensor and the gateway; and
- the duty cycle.

**Figure 17:**
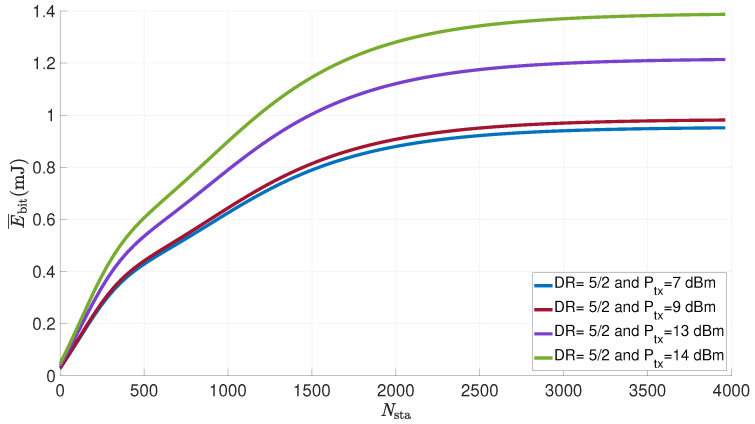
Average energy consumed per useful bit by a LoRa sensor for various transmit powers vs. number of nodes in the network (FPayload=50 bytes; NR=8; d=1km; dc=1%).

Figure 17 shows the evolution of the average energy consumed per useful bit by a LoRa sensor versus the number of sensors in the network for various transmit powers. The transmit power varies between 7 and 14 dBm, the number of transmission attempts has been set to eight and the data frame payload to 50 bytes. The duty cycle is set to 1% and the node is considered to be at a distance of 1 km from the gateway.

We can see that the average energy consumed per useful bit increases with the number of sensors, until it reaches a limit. Note that for one sensor in the network, the success transmission probability is close to 1 (● 1). Then, the sensor makes a single attempt and consumes energy equal to 19.56 mJ (see Table 9 for DR5), which corresponds to an energy per useful bit of 0.048 mJ, as shown in Figure 17. On the contrary, when the number of sensors is very high, for example, 4000, the failed transmission probability is close to 1 (▲ 4). In this case, the node makes eight attempts with failure, and consumes energy equal to 562.06 mJ (see Table 9 for DR5, DR4, DR3 and DR2), which corresponds to an energy per useful bit of 1.4 mJ, as shown in Figure 17. The threshold is obtained when the number of nodes is greater than 2000. Beyond this value, the number of collisions is too high, the probability of success is too low and the node uses the maximum number of allowed retransmissions without succeeding in transmitting the data. The energy consumed by the node also increases with the transmission power. We can conclude that a good Adaptive Data Rate (ADR) algorithm should take into account the distance in order to optimize the transmission power in each DR.

**Figure 18:**
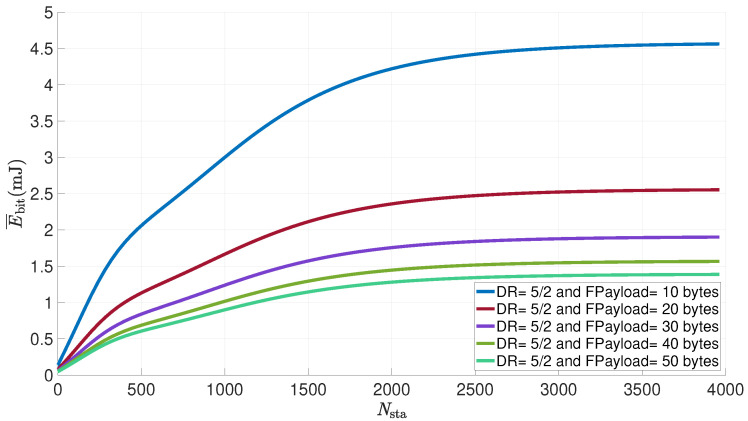
Average energy consumed per useful bit by a LoRa sensor for various payload sizes vs. number of nodes in the network (NR=8; $P_{\mathrm{tx}}=14\;\mathrm{dBm}$; d=1km; dc=1%).

Figure 18 shows the evolution of the average energy consumed per useful bit by a LoRa sensor versus the number of sensors in the network for various payloads transported in the data frames. The data frame payload varies from 10 to 50 bytes, the transmission power is set to 14 dBm, the number of retransmissions to eight, the distance between the node and the gateway to 1 km and the duty cycle is set to 1%.

As seen in Figure 17, the energy consumed per useful bit increases with the number of sensors until it reaches a threshold above 2000 nodes. The energy consumed also changes according to the data frame payload, with a ratio of one third between a load of 10 bytes and a load of 50 bytes. The growth of the payload reduces the amount of energy consumed per useful bit but also increases the transmission time, and thus the probability of collisions, as seen in (10).

**Figure 19:**
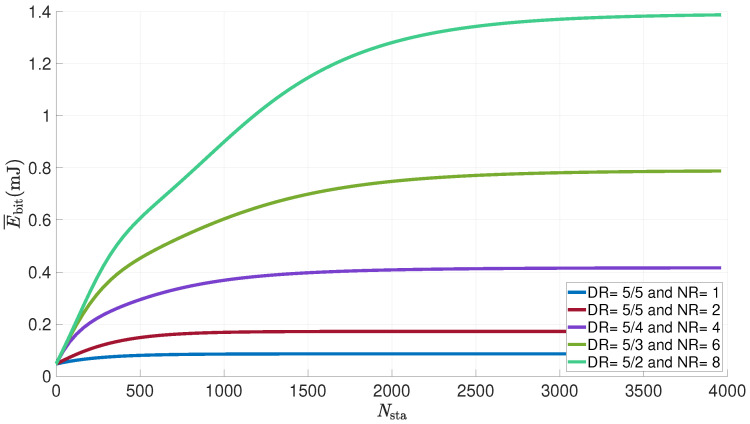
Average energy consumed per useful bit by a LoRa sensor for various numbers of attempts vs. number of nodes in the network (FPayload=50 bytes; $P_{\mathrm{tx}}=14\;\mathrm{dBm}$; d=1km; dc=1%).

Figure 19 shows the evolution of the average energy consumed per useful bit by a LoRa sensor versus the number of sensors in the network for various numbers of attempts allowed. The payload is set to 50 bytes, the transmit power to 14 dBm and the duty cycle to 1%; the DR changes according to the number of attempts and the number of attempts varies between one and eight.

Once again, we can see that the energy consumed evolves with the number of sensors, reaching a maximum when there are more than 2000 devices. The number of attempts also has an impact on the consumed energy. The equipment consumes twice as much energy for eight attempts than it does for six and twice as much energy for four attempts as for two. The DR decreases with additional retransmissions, which increases the energy consumed per useful bits. Thus, each additional attempt increases the amount of energy in a ratio equal to the double.

**Figure 20:**
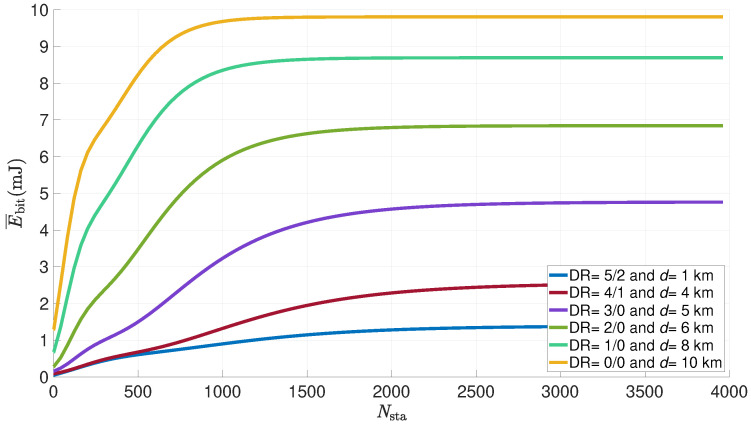
Average energy consumed per useful bit by a LoRa sensor for various distances vs. number of nodes in the network (FPayload=50 bytes; NR=8; $P_{\mathrm{tx}}=14\;\mathrm{dBm}$; dc=1%).

Figure 20 shows the evolution of the average energy consumed per useful bit by a LoRa sensor versus the number of sensors in the network for various distances between the node and the gateway. The DR changes according to the distance, the payload is 50 bytes, the power is 14 dBm, the number of attempts is set to 8 and the duty cycle to 1%.

In this figure, we can see the same evolution as before. The energy consumed per useful bit also evolves proportionally with the distance between the sensor and the gateway, for reasons that are similar to the number of retransmissions. An ED that is located more than 10 km away will transmit only with a DR0, an ED that is located less than one kilometer away will use DR 5 to 2. The threshold is also reached more quickly as the distance increases, at about 750 for a distance of 10 km and 2000 for a distance of 1 km. It can be concluded that, in order to optimize the energy consumption of the entire network, the transmission of the farthest nodes should be favored over the closest nodes.

**Figure 21:**
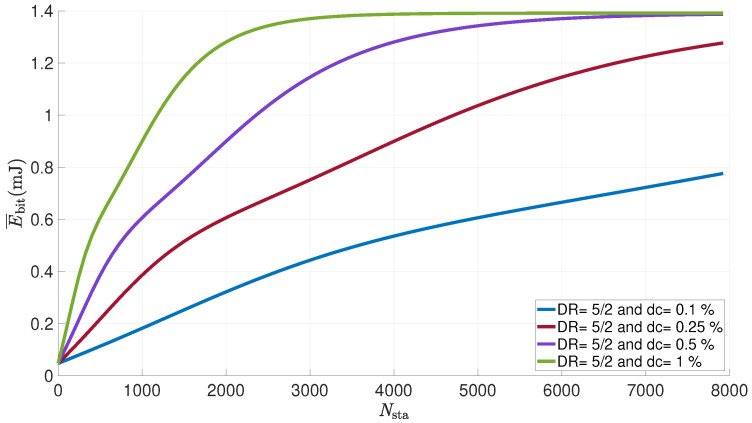
Average energy consumed per useful bit by a LoRa sensor for various duty cycle vs. number of nodes in the network (FPayload=50 bytes; NR=8; $P_{\mathrm{tx}}=14\;\mathrm{dBm}$; d=1km).

Figure 21 shows the evolution of the average energy consumed per useful bit by a LoRa sensor versus the number of sensors in the network for different values of the duty cycle. The number of attempts is set to eight, the payload to 50 bytes, the distance to 1 km and the power is set to 14 dBm.

Here again, we can observe that the energy consumed by a device increases with the number of nodes in the network. Collisions, and therefore the probability of retransmissions, follow exactly the same evolution. They cause an increase in the energy consumption per useful bit. A saturation can also be seen when the number of sensors exceeds 2000 devices. The probability of a successful transmission becomes very low and the number of collisions too high for the sensor to correctly transmit its data without the need for multiple attempts. The duty cycle also has an impact on the energy consumption. The number of sensors at which the saturation of the consumption is reached evolves with this ratio. For a value of 1%, the threshold is reached from 2000 nodes; for a value of 0.5%, the same threshold is reached from 5000 nodes; for a value of 0.25%, from 8000 nodes and beyond for a duty cycle of 0.1%.

From all these curves, it would be possible to optimize the energy consumption of the sensor network by a precise adjustment of the transmission parameters. These would be based on the use of the MAC commands that could allow us to provide the end devices with a configuration for the next transmission attempt. The ADR mechanism allows the network server to modify the DR and transmission power of the device; other MAC commands are used to request a change of the aggregated transmit duty cycle, times in idle state, channel skew or device status. Data aggregation would be the first solution. It would make it possible to decrease the duty cycle and increase the size of the data frames, which would have the effect of decreasing the energy consumption per useful bit and thus minimizing the number of transmissions and therefore transmission failures. The second solution would be to adapt the number of allowed attempts to the situation of the equipment and its environment. Sensors close to the gateway generally use a lower DR, so they consume less energy. The solution could be to allow them to make more attempts on channels with a higher sensor density.

## 5. Conclusions

In this paper, a refined energy consumption model based on in-situ measurements is provided for a LoRaWAN node. This improved model takes into account the number of nodes in the network, the collision probability that depends on the density of sensors and the number of retransmissions. In-situ measurements are made through a complete LoRaWAN testbed that includes the network server. The results allow us to characterize the node energy consumption and the time spent on each LoRaWAN communication step.

The simulation results show the influence of the number of nodes in a LoRaWAN network on the energy consumption of a node. The energy consumption also depends on classical parameters such as the transmission power, the load of the data frame, the number of retransmissions, the distance and the duty cycle. In all cases, the energy consumed per useful bit reaches a limit value that depends on the total number of nodes in the network. This demonstrates that the number of sensors that can be integrated into a LoRaWAN network is limited due to the probability of collision.

## Figures and Tables

**Table 1:** LoRa main features.

Parameter	Value
Modulation	CSS
Radio spectrum	US 915–EU 868
Data Rates	300 bps–37.5 kbps
Latency	500 ms
Bandwidth	(125, 250, 500) kHz
RSSI	−120 to −30 dBm
Range	5 km (urban)–15 km (rural)
Topology	star
Number of nodes	theoretically infinite

**Table 2:** LoRa DR mode characteristics in EU.

DRi	*i*	${\mathrm{SF}}_{i}$	BW	CR	Max MAC PL	Data Rate
DR5	5	7	125 kHz	4/5	250 bytes	5.47 kbps
DR4	4	8	125 kHz	4/5	250 bytes	3.125 kbps
DR3	3	9	125 kHz	4/5	123 bytes	1.76 kbps
DR2	2	10	125 kHz	4/5	59 bytes	0.98 kbps
DR1	1	11	125 kHz	4/6	59 bytes	0.44 kbps
DR0	0	12	125 kHz	4/6	59 bytes	0.25 kbps

**Table 3:** RF Sensitivity for the SX1272 devices.

DRi	*i*	${\mathrm{SF}}_{i}$	RF Sensitivity
DR5	5	7	−124 dBm
DR4	4	8	−127 dBm
DR3	3	9	−130 dBm
DR2	2	10	−133 dBm
DR1	1	11	−135 dBm
DR0	0	12	−137 dBm

**Table 4:** LoRa Frame Size.

Frame	Preamble	H1	H2	H3+FPayload	CRC
data	12.25 symb	20 bits	5 bytes	58 bytes	16 bits
ACK	12.25 symb	20 bits	5 bytes	8 bytes	-

**Table 5:** Currents consumed in different operating states.

Current			Times	
Parameter	Theoretical	Experimental	Parameter	Experimental
$I_{\mathrm{txwu}}$		2.268 mA	$t_{\mathrm{txwu}}$	1.722 ms
		21.86 mA (+3 dBm)		
	18 mA	22.36 mA (+7 dBm)		depends on
$I_{\mathrm{tx}}$		23.53 mA (+9 dBm)	$t_{\mathrm{tx},i}$	DR mode
($P_{\mathrm{TA}}$)		31.37 mA (+12 dBm)		(Table 6)
	28 mA	32.63 mA (+13 dBm)		
		39.43 mA (+14 dBm)		
$I_{\mathrm{txoff}}$		2.072 mA	$t_{\mathrm{txoff}}$	0.3 ms
$I_{\mathrm{id}}$	1.5 μA	123.4 μA	$t_{\mathrm{id}1}$	1 s
$I_{\mathrm{rxwu}}$		1.996 mA	$t_{\mathrm{rxwu}}$	9 ms
			$t_{\mathrm{ack},i}$	depends on
$I_{\mathrm{rx}}$	10.5 mA	10.76 mA	or	DR mode
			$t_{\mathrm{pre},i}$	(Table 6)
$I_{\mathrm{rxoff}}$		2.033 mA	$t_{\mathrm{rxoff}}$	0.3 ms
$I_{\mathrm{id}}$	1.5 μA	123.4 μA	$t_{\mathrm{id}2,i}$	$t_{w2}-t_{\mathrm{id}1}-t_{\mathrm{rx}1,i}$
$I_{\mathrm{rxwu}}$		1.86 mA	$t_{\mathrm{rxwu}}$	9 ms
			0 or	depend of
$I_{\mathrm{rx}}$	10.5 mA	11.12 mA	$t_{\mathrm{ack},0}$ or	DR mode
			$t_{\mathrm{pre},0}$	(Table 6)
$I_{\mathrm{rxoff}}$		2.054 mA	$t_{\mathrm{rxoff}}$	0.3 ms

**Table 6:** Data and ACK frame durations.

	Theoretical			Experimental		
DR*i*	$t_{\mathrm{tx},i}$	$t_{\mathrm{ack},i}$	$t_{\mathrm{pre},i}$	$t_{\mathrm{tx},i}$	$t_{\mathrm{ack},i}$	$t_{\mathrm{pre},i}$
DR5	118 ms	41.2 ms	8.2 ms	117.9 ms	39.8 ms	5 ms
DR4	215.6 ms	82.4 ms	16.4 ms	204.95 ms	78 ms	10 ms
DR3	390.1 ms	144.4 ms	32.7 ms	371 ms	140 ms	21 ms
DR2	698.4 ms	288.7 ms	65.5 ms	695 ms	290 ms	41 ms
DR1	1.708 s	626.7 ms	131 ms	1.675 s	612 ms	82 ms
DR0	3.219 s	1.253 ms	262.1 ms	3.195 s	1.134 s	230 ms

**Table 7:** Probabilities of an end-device using a specific SF.

${\mathrm{SF}}_{i}$	7	8	9	10	11	12
$p_{i}$	0.19	0.08	0.1	0.14	0.2	0.28

**Table 8:** Total time spent by the node in a communication phase for the various situations.

	DR5	DR4	DR3	DR2	DR1	DR0
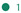	1.17 s	1.309 s	1.545 s	1.998 s	3.346 s	5.484 s
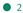	3.382 s	3.480 s	3.654 s	3.963 s	4.972 s	6.746 s
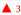	3.382 s	3.480 s	3.654 s	3.963 s	4.972 s	6.746 s
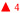	2.53 s	2.628 s	2.802 s	3.111 s	4.12 s	5.632 s

**Table 9:** Total energy consumed by the node in a communication phase for the different situations.

	DR5	DR4	DR3	DR2	DR1	DR0
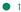	19.56 mJ	35.04 mJ	62.28 mJ	111.75 mJ	268.45 mJ	507.81 mJ
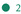	70.06 mJ	85.52 mJ	112.72 mJ	162.13 mJ	318.68 mJ	557.88 mJ
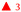	70.06 mJ	85.52 mJ	112.72 mJ	162.13 mJ	318.68 mJ	557.88 mJ
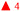	35.2 mJ	49.53 mJ	75.3 mJ	121.0 mJ	268.26 mJ	490.67 mJ
